# Hypopigmented Burn Scar Successfully Repigmented With Noninvasive Topical Bimatoprost Treatment

**DOI:** 10.7759/cureus.72402

**Published:** 2024-10-25

**Authors:** Kristin N Slater, Reem Kashlan, Geoffrey Potts

**Affiliations:** 1 Internal Medicine, Howard University Hospital, Washington DC, USA; 2 Dermatology, Wayne State University, Detroit, USA

**Keywords:** bimatoprost, burn, grease burn, hypopigmentation, hypopigmented scar, scar, topical bimatoprost

## Abstract

Burns can be disfiguring, causing noticeable pigmentary changes and scarring, which can be psychologically distressing. Scarring from burns can be notoriously difficult to treat, often requiring invasive, painful treatments. Here, we document the first reported case of successful improvement of a hypopigmented scar from a burn treated with topical bimatoprost 0.03% ophthalmic solution alone. We are hopeful that this will give a noninvasive option for patients who are looking to improve hypopigmented scars.

## Introduction

Burns can leave disfiguring scarring with noticeable pigmentary changes that are difficult to treat [1]. There are limited treatments to improve the cosmetic appearance of hypopigmented scar tissue [1]. This can cause significant psychological stress in patients [2]. Treatment modalities that report improvement in scar hypopigmentation include laser treatment combined with prostaglandin analogs [3] and microdermal grafting [4]. Each of these options requires invasive treatments to promote pigmentary improvement in hypopigmented scars. To our knowledge, there are no reported cases of burn scar hypopigmentation treated with topical bimatoprost 0.03% ophthalmic solution alone. Today, we report significant improvement in bilateral hypopigmented burn scars of the arms within three months of treatment with topical bimatoprost 0.03% ophthalmic solution, highlighting a potentially powerful noninvasive therapeutic option for hypopigmented scars.

## Case presentation

**Figure 1:**
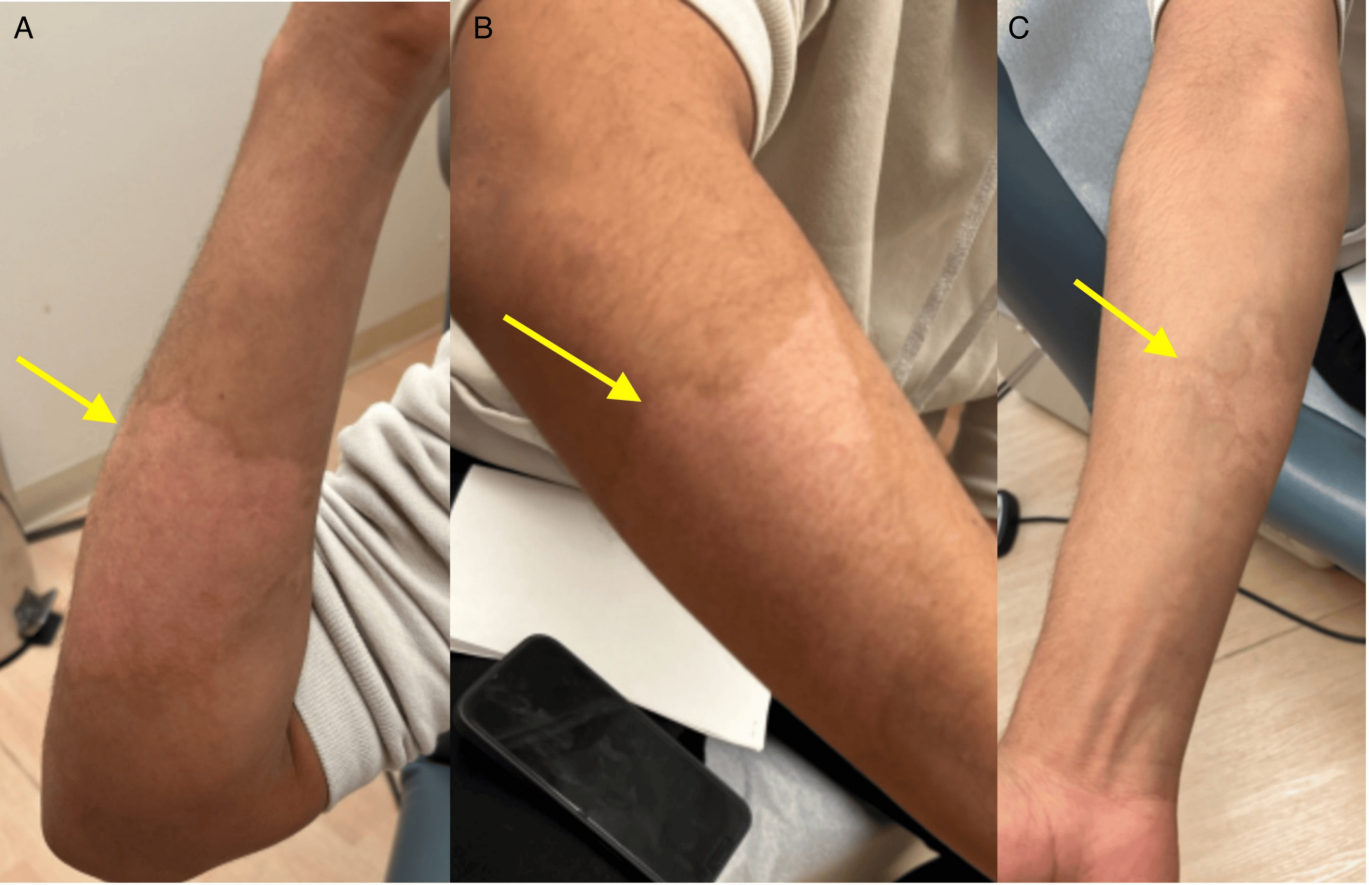
Ill-defined hypopigmented plaques on the right arm. A: Ill-defined hypopigmented plaque on the right lateral forearm. B: Ill-defined hypopigmented plaque on the right dorsal forearm. C: Ill-defined hypopigmented plaque on the right ventral forearm.

**Figure 2:**
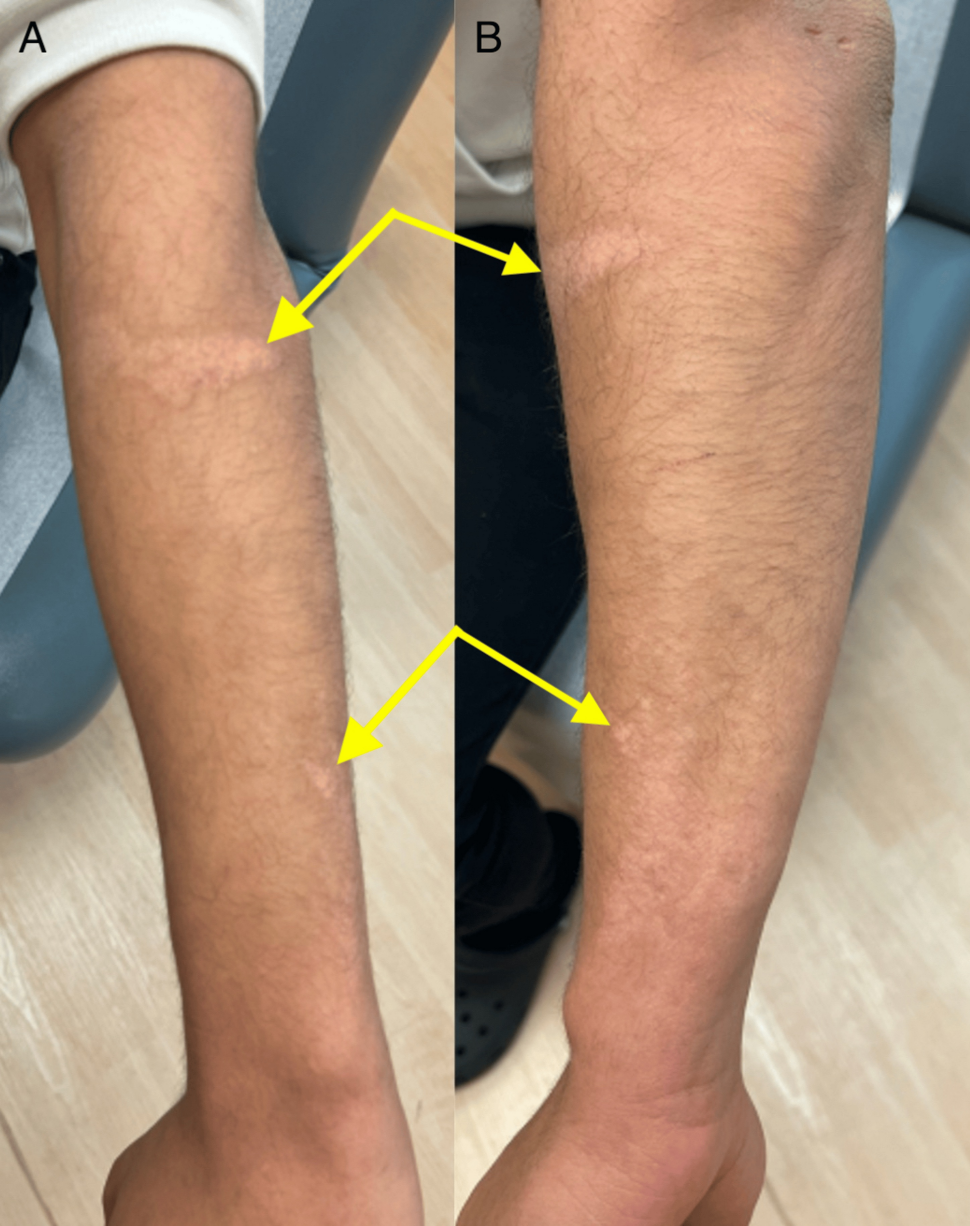
Ill-defined hypopigmented plaques on the left arm. A: Ill-defined hypopigmented plaques on the left dorsal forearm. B: Ill-defined hypopigmented plaques on the left lateral forearm.

**Figure 3:**
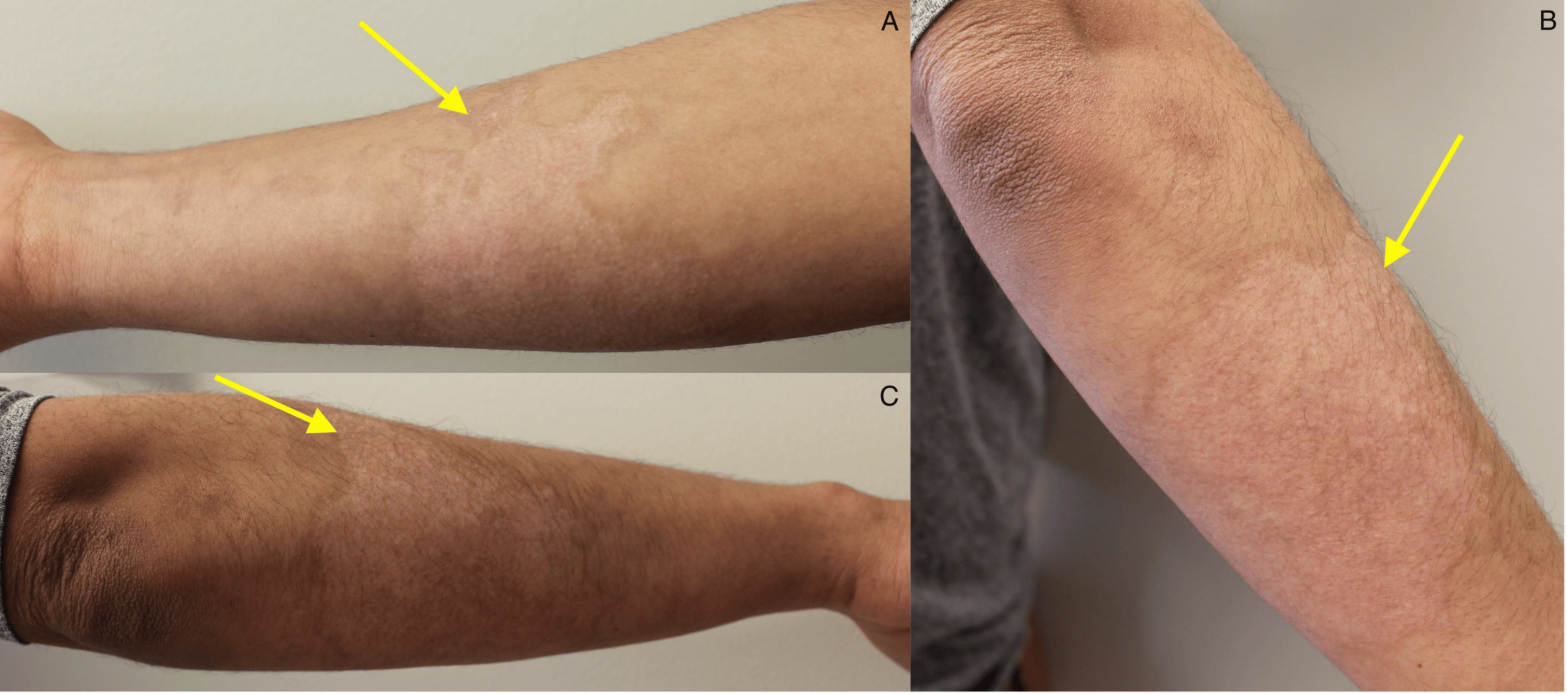
Improved pigmentation on the right arm. A: Improved pigmentation of the right ventral forearm. B: Improved pigmentation of the right dorsal forearm. C: Improved pigmentation of the right dorsal forearm.

**Figure 4:**
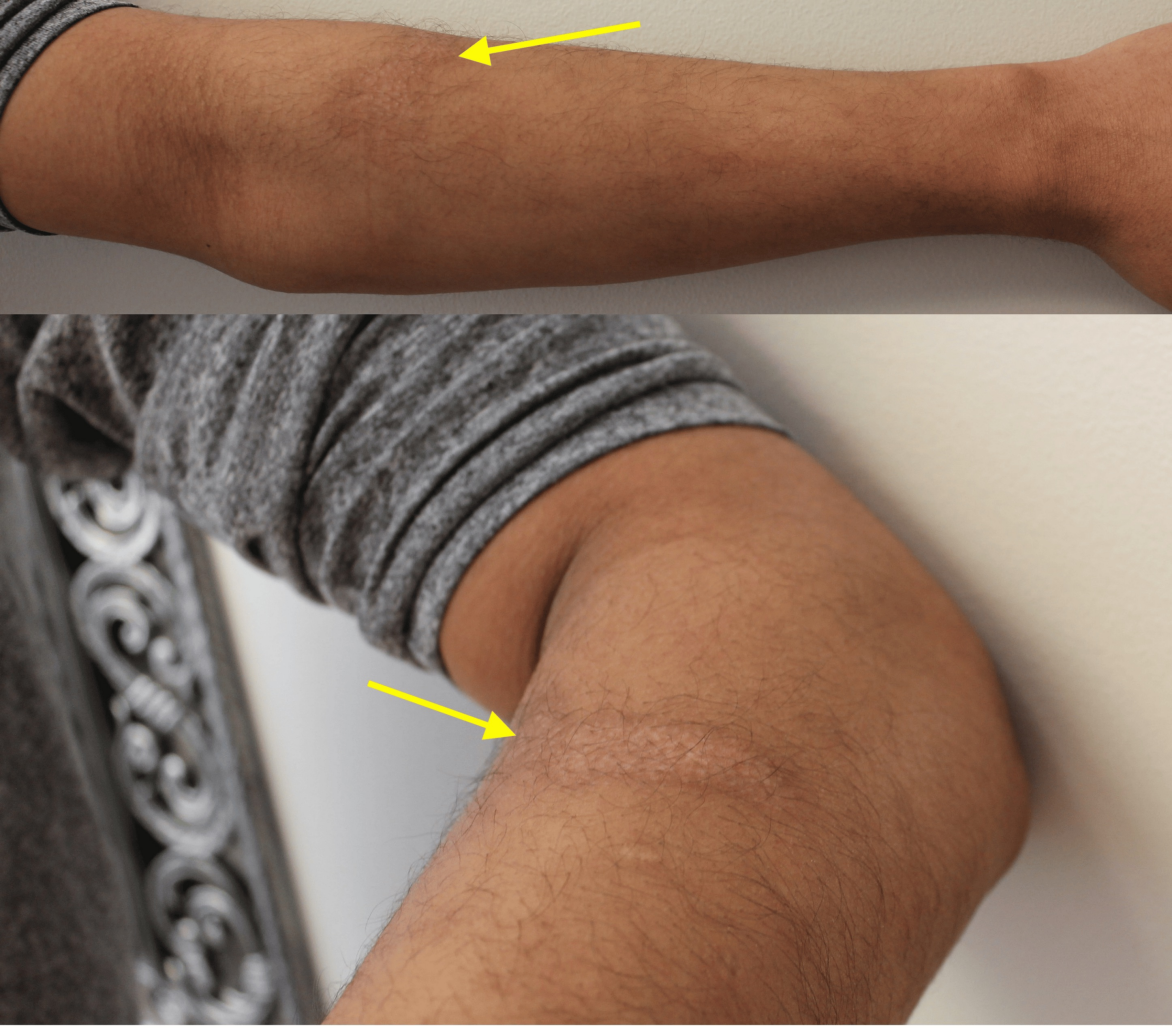
Improved pigmentation on the left arm. A: Improved pigmentation of the left dorsal arm. B: Improved pigmentation of the left lateral dorsal arm.

A 21-year-old male presented to the clinic with hypopigmented burn scars from a grease burn attained at work six months prior. He had no personal medical history and was on no medications. Physical examination showed ill-defined hypopigmented plaques on the bilateral arms (Figures 1, 2). There were no symptoms associated with the patient’s scars. He was started on topical bimatoprost 0.03% ophthalmic solution applied once daily to the hypopigmented scars and scheduled for a follow-up in three months. At the patient’s three-month follow-up, significant improvement in the hypopigmentation of his scars was seen (Figures 3, 4). The patient was instructed to discontinue topical bimatoprost 0.03% ophthalmic solution and follow up as needed. This case documents the first known case of a non-invasive topical treatment producing notable improvement in the appearance of hypopigmented burn scars.

## Discussion

Scars can be disfiguring, psychologically distressing [2], and difficult to treat [1]. Promising treatment modalities are invasive and include laser treatment combined with prostaglandin analogs [3] and microdermal grafting [4]. To date, we are only aware of one reported case of topical bimatoprost 0.03% solution with the combined use of microneedling and tacrolimus 0.1% ointment to improve the appearance of a hypopigmented burn scar [5]. In this case, microneedling with bimatoprost 0.3% solution was used over the span of one year with the frequency of once-a-month microneedling for six months, with an additional two sessions performed three months apart following the initial six sessions for a total of eight sessions [5]. In this report, tacrolimus 0.1% ointment was used daily throughout the year of microneedling treatments and for an additional four months following the treatment [5].

Topical bimatoprost has been successfully used to treat hypopigmentation caused by vitiligo as well [6]. Bimatoprost, a prostaglandin F2 alpha analog, is used as a treatment for hypotrichosis and open-angle glaucoma with the known side effect of hyperpigmentation [5-7]. As a prostaglandin F2 alpha analog, bimatoprost binds prostaglandin F receptors present on melanocytes, stimulating melanocyte dendricity and increasing tyrosinase activity [7]. This activity is thought to cause an increase in melanogenesis and melanocyte proliferation with corresponding hyperpigmentation through the inflammatory response and prostaglandin release [5,7].

Our case documents the first reported case of topical bimatoprost 0.03% ophthalmic solution used to improve the appearance of hypopigmented burn scars without any additional invasive techniques, such as microneedling [5] or laser treatment [3]. Bimatoprost 0.01% solution and tacrolimus 0.1% ointment were compared in patients with facial vitiligo, which showed comparable repigmentation results between the two topical treatments with the twice-a-day application over a 12-week duration [6]. We saw significant improvement in the hypopigmentation of the burn scarring on our patient’s bilateral arms within three months of treatment with topical bimatoprost 0.03% solution used once daily. This noninvasive treatment has the potential to increase treatment options for patients with hypopigmented scarring who cannot or would not like to undergo invasive treatments. Using bimatoprost 0.03% solution alone without invasive adjunctive therapies is not only more tolerable from a pain perspective but is also a more cost-effective, one-step option for patients looking to improve scar appearance in hypopigmented scars.

## Conclusions

To our knowledge, this is the only case to date documenting the successful use of topical bimatoprost 0.03% solution alone to treat hypopigmented burn scars. This treatment was both noninvasive and successful in improving the appearance of our patient’s hypopigmented burn scars. We hope that this novel finding can expand treatment options for hypopigmented scars, alleviating the psychological burden for scar patients.
